# Data on behavior and environmental impact of compostable packaging materials in full-scale industrial composting conditions

**DOI:** 10.1016/j.dib.2024.111102

**Published:** 2024-11-06

**Authors:** Emmanuelle Gastaldi, Felipe Buendia, Paul Greuet, Sandra Domenek

**Affiliations:** a Fondation AgroParisTech, Chaire CoPack, UMR SayFood, 91120 Palaiseau, France; b UMR IATE, Université Montpellier, INRAE, L'institut Agro Montpellier, 2 place Viala, 34000 Montpellier, France; c Université Paris-Saclay, INRAE, AgroParisTech, UMR SayFood, 91120 Palaiseau, France

**Keywords:** Composting process, Biodegradation, PLA, Thermoplastic starch, PHA, Fragmentation kinetics, Polymer morphology, Life cycle assessment

## Abstract

The dataset reports the impact of incorporating commercial compostable plastics into a full-scale open-air windrow composting process using household-separated biowaste. Two batches were prepared from the same biowaste mixture: one as a control and the other with 1.28 wt% of certified compostable plastics. The degradation of the materials was monitored over four months by regular sampling, which matched the industrial composting duration. The final compost was evaluated for agronomic quality and safety. Life-cycle assessment was performed based on data collected on process resource usage. The dataset includes an extensive review of full-scale composting experiments, raw and processed data on the composting process, biodegradation of the materials, disintegration kinetics, and the evolution of morphological parameters of the plastics. Industrial-scale data are very rare and can be compared with lab-scale data to assess the differences in compostable material behavior due to scaling up the process.

Specifications Table

**Table:** 

Subject	Waste Management and Disposal
Specific subject area	The biodegradation performance of representative commercial compostable materials in a full-scale open-air windrow composting process at industrial scale was evaluated.
Type of data	Table, Image, Graph, Figure Raw and analyzed
Data collection	Lab-scale data on biodegradation: manual measurement by capture of CO_2_ and titration Industrial-scale data of the composting experiment: manual measurement of temperature, humidity, composting yield, photographs using a mobile phone Compost quality and safety: external laboratory using standard procedures Mass loss of materials: manual sieving and weighting Morphological analysis of fragments: thermogravimetry, differential scanning calorimetry, size-exclusion chromatography, Fourier-transform infrared spectroscopy, scanning electron microscopy Life Cycle Assessment: on-site measurement of energy and water inputs, Ecoinvent database 3.0.1, Open LCA software v2
Data source location	The data and samples were collected in the composting platform Syndicat Centre Hérault (SCH), 34,800 Asprian, France. The sample processing and data generation was done in the partner laboratories: University of Montpellier, UMR IATE, 34,000 Montpellier and AgroParisTech, UMR SayFood, 91,120 Palaiseau. The data are stored in the repository “recherche.data.gouv.fr”.
Data accessibility	Repository name: Collection “Full scale composting experiment of compostable packaging” https://entrepot.recherche.data.gouv.fr/dataverse/full_scale_composting Datasets: General data of full scale composting experiment: doi: 10.57745/VQTQJ4 10.57745/VQTQJ4 Lab-scale biodegradation of compostable materials: doi: 10.57745/LEL1QE 10.57745/LEL1QE Photos of the composting experiment: doi: 10.57745/UFLG6G 10.57745/UFLG6G Monitoring of full scale composting experiment: doi: 10.57745/OSNDZH 10.57745/OSNDZH Evolution of granulometry of plastic fragments sampled during the composting experiment: doi: 10.57745/JNMRZI 10.57745/JNMRZI Photos of large plastic fragments sampled during the composting experiment and identified by FTIR: doi: 10.57745/XAASRZ 10.57745/XAASRZ Identification of large sampled plastics fragments during the composting experiment by FTIR doi: 10.57745/M3MYBU 10.57745/M3MYBU Evaluation of safety and quality of final compost: doi: 10.57745/UVBHCZ 10.57745/UVBHCZ Calorimetric analysis of plastics fragments sampled during full scale composting doi: 10.57745/DVBTEQ 10.57745/DVBTEQ Thermo-gravimetric analysis of plastic fragments sampled during the composting process doi: 10.57745/QVD8MX
	10.57745/QVD8MX Macromolecular weight averages of plastic fragments sampled during the composting experiment doi: 10.57745/9GGBUK 10.57745/9GGBUK Scanning electron microscopy data of plastic fragments sampled during the composting experiment doi: 10.57745/AZJS5N 10.57745/AZJS5N Life Cycle Assessment of the industrial composting process of biodegradable packaging: doi: 10.57745/5SAWNO 10.57745/5SAWNO Free access, License etalab 2.0
Related research article	Emmanuelle Gastaldi, Felipe Buendia, Paul Greuet, Zineb Benbrahim Bouchou, Anir Benihya, Guy Cesar, Sandra Domenek, Degradation and environmental assessment of compostable packaging mixed with biowaste in full-scale industrial composting conditions, Bioresoure Technology, 2024;400:130,670. 10.1016/j.biortech.2024.130670

## 1 Value of the Data

• The data fill a gap in open literature because they provide original and transparent information on the degradation of compostable plastics in an industrial environment.
• The data are useful to evaluate the performance of compostable plastics in a full-scale environment using curbside collected biowaste.
• Other researchers can use these results to compare with their own lab-scale findings and to design complementary experiments at the industrial scale.
• The acquired process data can be used for the validation of composting models.

## 2 Background

This Data-in-Brief article complements the findings from our original research article [1] and the freely accessible raw data. It includes an overview of the experiment setup, monitoring procedures, and results, along with a brief description of the methodology. The article presents raw data related to process monitoring, polymer degradation, safety and quality assessments, and life cycle assessment. The research aimed to evaluate the behavior of representative commercial compostable materials in a full-scale, open-air windrow composting process at an industrial site using curbside-collected, household-separated biowaste. Open data on full-scale composting experiments are very scarce. This dataset is unique as it is the first to simultaneously monitor composting processes with and without compostable plastics. To achieve this, the starting materials were divided into two batches: a control batch and another batch mixed with compostable plastics, both subjected to the same composting process in parallel. The data can be used for comparison or model validation.

## 3 Data Description

The dataset presented in this article is published in the data collection “Full scale composting experiment of compostable packaging”; https://entrepot.recherche.data.gouv.fr/dataverse/full_scale_composting. It includes raw and processed data were recorded during the composting experiment of compostable plastics at industrial scale. The following article describes the data, which can be found in the data collection.

1. **General data:** The dataset titled ``General data of full scale composting experiment (10.57745/VQTQJ4)'' includes comprehensive details about the composting experiment, including the coordinates and a description of the industrial composting facility. It also contains information on the test materials used and a review of relevant scientific literature that informed the development of the experimental strategy.
2. **Lab-scale composting test:** The dataset “Lab-scale biodegradation of compostable materials (10.57745/LEL1QE)” contains raw data from a lab-scale biodegradation experiment conducted under industrial composting conditions, following the standard EN 13432. This dataset includes measurements of the total carbon content of the test materials and the raw data from the laboratory experiment, such as temperature, humidity, and the amount of generated CO_2_.
3. **Monitoring of the industrial composting process:** The monitoring data for the industrial composting experiment are split into two datasets: “Photos of the composting experiment (10.57745/UFLG6G)” features photographs taken onsite at various stages of the composting process, while “Monitoring of full scale composting experiment (10.57745/OSNDZH)” contains raw data on compost parameters such as temperature, relative humidity, and self-heating tests, which were recorded by the composting platform. Regular sampling during the composting process led to the recovery and analysis of plastic fragments, with the dataset "Evolution of granulometry of plastic fragments sampled during the composting experiment (10.57745/JNMRZI)" reporting the quantity and size of these fragments at each sampling point. Larger recovered fragments were chemically identified via Fourier-Transform Infrared Spectroscopy (FTIR). The dataset “Photos of large plastic fragments sampled during the composting experiment and identified by FTIR (10.57745/XAASRZ)” contains images of these fragments, while “Identification of large sampled plastics fragments during the composting experiment by FTIR (10.57745/M3MYBU)” includes the corresponding FTIR spectra.
4. **Quality and safety evaluation:** The dataset “Evaluation of safety and quality of final compost (10.57745/UVBHCZ)” details measurements performed according to the NF U44-051:2006 standard for organic soil improvers. The safety parameters cover the detection of inert materials, impurities, viable pathogens (e.g., helminth eggs and Salmonella), polycyclic aromatic hydrocarbons (PAHs), and trace elements. The agronomic quality evaluation includes the measurement of total solids, organic matter, carbon, nitrogen, C/N ratio, pH, and concentrations of essential nutrients like phosphorus, potassium, calcium, magnesium, and sulfur. The phytotoxicity test assesses the compost's potential to inhibit the growth of two plant species (barley and watercress), while ecotoxicity is evaluated using the toxicity test on earthworms (*Eisenia fetida*) in compliance with NF ISO 11,268–1:2012. In addition, data of the immobilization test on microcrustaceans (*Daphnia magna*) performed according to ISO 6341:2012, using a liquid extract obtained following EN 12,457–2:2002 standards are reported.
5. **Morphological analysis of fragments:** The morphological changes in recovered plastic fragments were analyzed using various physico-chemical methods, and the raw data are distributed across four datasets. The changes in thermal stability were analyzed via Thermogravimetric Analysis (TGA) and raw data can be found in “Thermo-gravimetric analysis of plastic fragments sampled during the composting process (10.57745/QVD8MX)”. Variations in glass transition temperature and melting enthalpy were assessed using Differential Scanning Calorimetry (DSC), with the raw data provided in “Scanning electron microscopy data of plastic fragments sampled during the composting experiment (10.57745/DVBTEQ)”. Size-exclusion chromatography (SEC) was used to measure the molecular weight averages, with the chromatograms reported in “Macromolecular weight averages of plastic fragments sampled during the composting experiment (10.57745/9GGBUK)”. Lastly, images of the fragments taken by Scanning Electron Microscopy (SEM) are available in “Scanning electron microscopy data of plastic fragments sampled during the composting experiment (10.57745/AZJS5N)”.
6. **Life cycle assessment:** The LCA was conducted in accordance with ISO 14040 and 14044 (2006) guidelines, utilizing the OpenLCA v2 software and the Ecoinvent® 3.0.1 database. The dataset “Life Cycle Assessment of the industrial composting process of biodegradable packaging (10.57745/5SAWNO)” includes both inventory data and the calculated environmental impact metrics.

## 4 Experimental Design, Materials and Methods

### 4.1 Literature review, test materials and testing site

For the design of the composting experiment, a literature review of former full-scale composting experiments was carried out using the database “Web of Science”. In total 8 articles were found which reported full-scale composting of biodegradable plastics in industrial environment or the large pilot scale (> 100 L compost) and presented in Table 1.

**Table 1:** Composting tests of biodegradable plastics in municipal waste treatment facilities.

Sample	Composting method	Result	Location	References
PLA	Turned windrow, samples in wooden boxes, T_max_ 55 °C	Gradual embrittlement, *M_w_* decrease, visual inspection shows complete disintegration in 30 d	Michigan, USA	[2]
PLA PHB and blends	Static open-air pile (T_max_ 60 °C) or in containers (T_max_ 64 °C); samples in metal cages with branches and leaves	Degradation monitored by *M_w_* decrease, sampling after 21 and 70 days, PHB degraded faster than PLA, blends increase PLA degradation, no information on disintegration or weight loss	Zabrze, Poland	[3]
PLA	Static open-air pile (T_max_ 60 °C) or in containers (T_max_ 64 °C); samples in metal cages with branches and leaves	Degradation monitored by *M_w_* decrease, analysis of Mw profiles shows autocatalytic degradation of PLA, after 70 d, samples are recovered, no information given on disintegration or weight loss	Zabrze, Poland	[4]
Compostable tableware PLA, PLA/fiber laminates, pressed fiber based dishes Kraft paper	Turned windrow (T_max_ above 60 °C), samples in mesh bags; anaerobic digestion (35 °C) followed by static pile; samples in mesh bags; static pile with forced aeration (60 °C), samples in mesh bags; In vessel composting (no mesh bags, 60 °C) followed by roofed static pile	Percent of samples in the mesh bags 10 or 20 vol%; Disintegration in turned windrow after 65 d: PLA cutlery 80 %, laminated PLA/fibers 100 %, Kraft control, fiber dishes < 10 %; anaerobic digestion after 49 d: no degradation of PLA, variable percentages of laminated and fiber based references between 0 and 80 %; In static pile after 50 d: 100 % except Kraft paper and some paper dishes; in vessel after 82 d: 100 % of all references; Weight loss 100 % for PLA references except anaerobic digestion, of fiber based materials variable, except for in vessel composting, 100 % weight loss of all samples, microbial activity enhanced by high concentration of samples	British Columbia, Canada	[5]
PLA, starch based blends	Pilot trial, biodegradable packaging sold to consumers, recovered through municipal waste collection, composting in windrow (no technical specs)	Biodegradable polymers recovery ca. 40 wt%, max. percentage in the compost 0.47 wt%, no negative effect in the composting plant but manual sorting leads to material loss, no changes in compost quality, no change in agricultural yield using the compost	Kassel, Germany	[6]
PLA tableware, starch-based bags, Kraft paper	Turned windrow (T_max_ 70 °C)	PLA disintegration 100 % after 7 weeks, disintegration of starch-based materials and Kraft paper between 80 and 90 % after 20 weeks; average germination index of PLA smaller than cellulose of Kraft paper, germination index of starch-based materials higher than cellulose or Kraft paper	Chico, USA	[7]
Cellulose derivatives, paper, cellulose nanofibers	200 L composter bin with continuous aeration with CO_2_ and temperature measurement, manual turning, T_max_ 70 °C, samples attached to plastic frames	Disintegration 100 % after 12 weeks for all references, except cellulose acetate, octanoate and palmitate which showed no degradation	Finland	[8]
PLA	180 L fermenter with CO_2_ measurement, T_max_ 75 °C,	5 % residual mass on a 2 mm sieve after 65 d, no impact on C/N ratio of final compost, no impact on germination and plant growth tests	Japan	[9]

PLA. polylactide or poly(lactic acid), PHB. poly(3-hydroxybuturate-co-3-hydroxyvalerate), Mw. macromolecular weight.

Based on this information, the technology of open-air turned windrow composting was selected. The full-scale composting experiment was carried out at the Syndicat Centre Hérault (SCH), an industrial composting facility situated in Aspiran, France (34,800). This composting site collects 76 rural municipalities, accounting for 80,000 inhabitants and a surface of 1100 km² (20 % of the surface of the Department Hérault, France). Many households in the area use compostable plastic bags for the biowaste collection. It receives approximately 3000 tons household separated biowaste per year. The green waste stream is collected from a network of waste collection centers and amounts around 6000 tons per year.

The Table 2 sums up the test materials which were mixed with the biowastes. It is reproduced from the main article [1] to ease the reading of the DIB article.

**Table 2:** Characteristics and amounts of the compostable materials included in the materials batch.

Material	Application	Polymer composition	TUV Austria label	Thickness max-min (µm)	Amount added (kg)	Initial conc. in biowaste (%)	Color
E1	Waste bin Bag	Polyester/starch	OK_H_	93–17	50	0.20	Green
E2	Fruit & Veg. Bag	Polyester/starch	OK_H_	45–12	10	0.04	Transparent
E3	Carrier Bag	Polyester/starch	OK_I_	174–48	65	0.26	Blue
E4	Fruit & Veg. Bag	Polyesters blend	OK_I_	37–9	30	0.12	White
E5	Film	Polyester ecoflex	OK_H_	180–22	30	0.12	Transparent
E6	Tray	PLA	OK_H_	429–381	65	0.26	Green
E7	Tray	PLA	OK_I_	406–325	20	0.077	Transparent
E8	Tray	Cellulose + polyester	OK_I_	450–430	30	0.12	White
E9	Coffee capsule	PHA	OK_I_	1200–720	20	0.079	Pink
E10	Coffee capsule	Starch blend	OK_I_	3089–850	2.5	0.01	White

OK_H_. label OK compost home for domestic composting conditions, OK_I_. label OK compost industrial for industrial composting conditions, Conc. concentration in 20 t of mixed biowaste, from [1].

### 4.2 Lab-scale biodegradation kinetics of test materials

The biodegradation of the different types of plastics was analyzed according to NF ISO 14855-1 (2012) for industrial composting conditions (58 °C, 50 % humidity) using a miniaturized set-up described by [10]. For that, samples were cut in pieces of 5 × 5 mm. Exactly 50 mg of equivalent carbon were introduced into 3 g of compost previously sieved at 2 mm. A volume of 2.7 mL of deionized water was added to obtain a humidity of 50 wt%. The carbon contents of the different samples were measured by elemental analysis (ThermoQuest NA 2500, CE Instruments Ltd, Wigan, UK). Experiments were done in a hermetic glass jar (1 L, Le Parfait, Villeurbanne, France) which enclosed three open polypropylene vials (60 mL) each. One vial contained 3 g of humid compost mixed with the sample samples, the second contained 15 mL of NaOH (0.2 M), which trapped the CO_2_ released by the microorganisms, and the third distilled water to keep the relative humidity inside the jar close to 100 %. The jars were hermetically closed and incubated in the dark at 58 ± 1 °C. At the selected time, the glass jars were opened to determine the amount of CO_2_ trapped by the NaOH solution by back titration of the carbonate ions. The latter were then precipitated by the addition of 5 mL of barium chloride solution (20 wt% in water), in each flask, in the presence of thymophthaleine (0.10 % in ethanol) and titrated by a HCl solution (0.1 M). The biodegradation tests included a positive control and a blank. A powder of pure cellulose (BE 600-10 TG grade) provided by Arbocel J. Rettenmaier & Söhne (Rosenberg, Germany), with a median apparent diameter (d50) of 18 µm, was used as positive control. For the blank, the experiment was conducted without adding any sample, to be able to measure the CO_2_ naturally produced by the compost and the CO_2_ present in the air of the glass jar. All experiments were performed in triplicate. The CO_2_ production of the blank was subtracted from the measurements. The percentage of biodegradation (%) was calculated using the following equation:

(1) $$Biodegradation=\frac{CO_{2,\text{sample}}-CO_{2,\text{blank}}}{CO_{2,\;\text{theoretical}}}\cdot 100$$

where CO_2,sample_ and CO_2,blank_ are the amounts of CO_2_ (mg) released in the test jar and in the blank control jar, respectively. CO_2,theoretical_ is the theoretical amount of carbon dioxide (mg) produced by the total oxidation of the carbon added by the test material. As required by the NF ISO 14855-1: 2012 standard, the validity of the test is obtained when cellulose (used as a positive control) reaches a biodegradation percentage higher than 70 % in less than 45 days.

The mineralization curves were modelled with Hill's equation as initially proposed by [11]:

(2) $$\text{Biodegradation}=\;\text{De}g_{\text{max}}\cdot \frac{t^{n}}{K^{n}+t^{n}}$$

Biodegradation is the percentage of degradation [%] at time *t* [d], Deg_max_ [%] the percentage of degradation at infinite time, *K* [d] the half-life time and *n* the curve radius of the sigmoidal function.

**Fig. 1:**
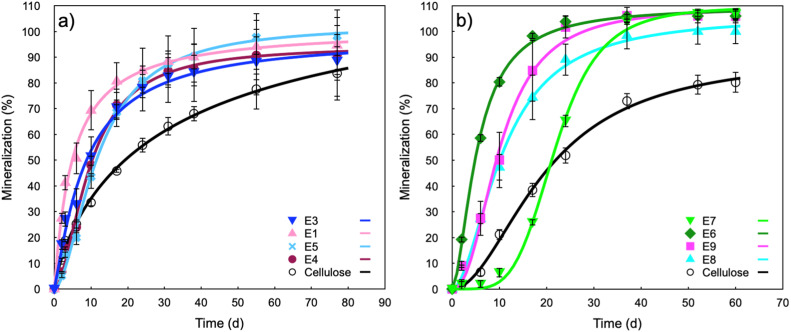
Biodegradation curves of the commercial test materials (reproduced ref.1): a) flexible films, b) rigid materials. The sample codes are explained in Table 2.

Fig. 1 shows the original data of the measured mineralization percentage of the different plastics, where the lines correspond to the modeling using Hill's equation. The modelling parameters of flexible and rigid materials are given in Table 3, Table 4, respectively.

**Table 3:** Hill parameters of biodegradation modeled curves of flexible materials in industrial composting conditions.

Sample	Hill parameters			
	Degmax (%)	*K* (days)	n	R²
Cellulose E1 E3 E4 E5	128.17 ± 10.28 101.51 ± 2.99 98.46 ± 0.13 94.46 ± 2.32 102.75 ± 2.39	33.46 ± 7.10 5.01 ± 0.41 8.55 ± 1.07 9.65 ± 0.52 11.80 ± 0.55	0.78 ± 0.05 1.03 ± 0.09 1.16 ± 0.13 1.78 ± 0.14 1.78 ± 0.11	0.998 0.997 0.993 0.996 0.997

**Table 4:** Hill parameters of biodegradation modeled curves of rigid materials in industrial composting conditions.

Sample	Hill parameters			
	Degmax (%)	*K* (days)	*n*	R²
Cellulose E6 E7 E8 E9	90.87 ± 3.48 109.65 ± 1.30 109.57 ± 1.75 106.19 ± 2.58 109.71 ± 3.00	19.74 ± 1.13 5.35 ± 0.18 21.87 ± 0.38 10.72 ± 0.49 10.09 ± 0.52	1.95 ± 0.15 1.69 ± 0.09 4.64 ± 0.36 1.85 ± 0.15 2.29 ± 0.26	0.998 0.999 0.998 0.998 0.995

### 4.3 Monitoring of the industrial composting process

The composting piles were constituted as described in [1]. During the composting process, which started the 26th September 2023, regular samples were taken at the moment of turning to monitor de biodegradation. The compost parameters (temperature and humidity) were monitored during the process. Watering and turning were carried out simultaneously twice a week for the first 3 weeks of the active thermophilic phase. Then frequency was then reduced to once a week. The humidity was controlled on a representative sample by drying in an oven at 103 ± 1 °C until constant weight. After monitoring, leachate water was used, if necessary (in absence of rainfall), to set back the moisture content to approximately 50 %. The process lasted 128 days. The degradation of the plastics was measured by regular sampling at the moment of windrow turning. The visual changes of the compost were documented with a smartphone camera. Table 5 shows pictures of the compost taken at each sampling time.

**Table 5:** Photos documenting the visual degradation of compostable plastics in the materials batch during the composting experiment.

The composition of the initial compost mixture and the composting yield after the composting process and screening are reported in Table 6.

**Table 6:** Composition of the control and compostable batches at the start of the composting process, mass of the different fractions obtained after screening, screening and composting yields.

	Control batch	Control batch normalized H%^a^	Materials batch	Materials batch normalized H%^a^
***Composition before composting***				
Biowaste collected (t)	20.02		20.12	
*Sorting errors removed (t)*	*0.25*		*0.30*	
Plant shredding (t)	2.502		2.515	
Grass cuttings (t)	2.502		2.515	
Green waste/biowaste (%)	25.31		25.37	
Compostable items added (t)	–		0.322	
Total treated (t)	24.74	24.74	25.17	25.17
***Composition after 128-days composting***				
Total mass (t)	9.78	9.78	10.94	10.11
***Composition after screening***				
Coarse fraction (>80 mm) (t)	1.12	1.12	1.09	1.01
Medium fraction (12–80 mm)	3.20	3.20	3.36	3.10
Fine fraction (<12 mm)	5.46	5.46	6.50	6.01
Total compost output	5.46		6.50	
***Yield***				
Screening yield (%)^b^		55.8		59.4
Composting Yield (%)^c^		27.2		28.7

a Normalized mass calculated after considering a water content at 38.38 % for both batches.

b Screening yield = screening rejects (= coarse + medium fractions)/total mass after composting.

c Composting Yield = total compost output (fine fraction) / total initial mass sent for composting.

The sampling of the compost during the process was done using the standardized method NF EN 12579:(12579:2013, 2013). For a batch of compost (max 580 m³) it recommends taking 12 elementary samples, each weighing 1 kg, from various locations. The quartering method described in ASTM C702/C702M:2018 (C702/C702M:2018, 2018) was used to reduce the sample size without creating a systematic bias due to product heterogeneity and obtain a representative final sample of around 10 kg per batch and per sampling time. Table 7 shows pictures of an example of the screening of the different samples to measure the mass loss. The screened plastics were recovered with the help of tweezers and the weighted without further cleaning. The measured quantities of the different materials are given in the Table 8.

**Table 7:** Analysis of granulometric distribution of sampled compost (example 18 d).

**Table 8:** Recovered plastics concentration over time.

Fragment size (mm)		Fragment concentration (%)						
	Time (d)	0	18	30	45	59	107	128
All	>10 mm	1.286	0.347	0.830	0.784	0.371	0.0623	0
	5 < 10 mm		0.169	0.112	0.038	0.017	0.0183	0.0106
	5 < 2 mm		0.149	0.118	0.062	0.058	0.0148	0.0190
	2 < 1 mm		0.018	0.013	0.006	0.008	0.0009	0.0031
Colored	>10 mm	0.599	0.075	0.0931	0.055	0.004	0	0
	5 < 10 mm		0.109	0.054	0.023	0.010	0.0078	0.0052
	5 < 2 mm		0.124	0.102	0.056	0.051	0.0124	0.0134
	2 < 1 mm		0.014	0.011	0.005	0.008	0.0008	0.0020

Specifically in the fraction of large fragments, the recovered quantities fluctuated. Although the biowaste was manually cleaned at the start of the experiment, sorting errors cannot be entirely ruled out. To account for this, the polymer type of large fragments was manually identified using Fourier-Transform Infrared Spectroscopy (FTIR) with a Perkin Elmer Vertex IR, operated in ATR mode via the OPUS software. The resolution was set at 4 cm⁻¹, with a scanning range of 4000–800 cm⁻¹. Each acquisition consisted of 50 scans, with spectra displayed in absorbance mode. Chemical identification was conducted by comparing the spectra with reference spectra of the initial plastic samples, accepting a correlation coefficient of ≥ 90 %. Table 9 provides examples of these identifications. Data for all measured FTIR spectra across different sampling times are available in the open data repository.

**Table 9:** Example of identification of sampled polymer fragments at day 45.

### 4.4 Evaluation of compost quality

The safety and agronomic quality of the screened final compost from both the materials and control batch were evaluated. Both batches demonstrated equally high agronomic value, complying with the existing quality standard NF U44-051:2006 and meeting the requirements for organic agriculture. No acute ecotoxicity was observed. The findings are presented in [1], and the raw data can be accessed in the open data repository.

### 4.5 Morphological studies of plastics fragments

The morphological studies of the plastics fragments were carried out using the colored materials, which were used as tracers of the degradation of a given polymer family (Table 2. E3 – blue, E6 – green, E9 – pink). Their coloration eased the recovery and identification of the fragments. The surface properties, fragmentation due to degradation and the microbial attachment to the surface were be observed in the scanning electron microscopy (SEM). SEM observations in backscattering mode on a metalized free surface were performed using a Desktop SEM (Phenom ProX, Fondis Bioritech, France) with an acceleration voltage ranging between 5 and 10 kV. Samples were directly mounted on stub using carbon conductive tape and then coated with Gold/Palladium during 45 s at 20 mA (4 nm thick) by ion sputtering (Mini Sputter Coater SC7620, Quorum Technologies Ltd, UK). The images are shown in Table 10.

**Table 10:** SEM images of sampled plastics fragments during the composting process.

The evolution of the macromolecular mass averages and the dispersity of the colored fragments during the composting process was analyzed with Size-Exclusion Chromatography (SEC). It was performed using a Gilson pump (France) coupled to a Waters autosampler and refractometric index detector (Waters, France). The separation was carried out on a system consisting of a guard column (PLgel 5 µm) and three columns (two columns PLGel 5 µm MIXED-C and one column PLgel 3 µm MIXED E, Agilent Technologies) maintained at 40 °C in a column oven (Waters, France). The flow rate of the CHCl_3_ was 1 mL/min. The calibration curve was established using three standard kits (EasiVials, 2 mL) containing each four narrow polystyrene standards of molecular weight between 4.69·10^3^ and 5.68·10^6^ g/mol (Agilent Technologies). Data treatment was done using Empower 3 software (Waters). Samples were prepared by dissolution of the uncleaned solids in dried CHCl_3_ (app. 20 mg/mL) at room temperature or after heating at 40 °C without stirring at least 4 h. The supernatant was sampled after sedimentation of the insoluble phase and filtered before analysis with the help of 0.45 µm Teflon syringe filters. Because of the biodegradation of the materials during the process, the less and less sample mass was available. If enough sample available, the preparations were done several times (at least in duplicate). Table 11 summarizes the data.

**Table 11:** Evolution of the macromolecular weight average of the colored materials during the composting process using SEC.

Fragment size (mm)		Macromolecular weight average (Dalton)			Dispersity (-)		
	Time (d)	0	18	59	0	18	59
PHA (E9 - pink)	outside	162,788±10,000	73,680±2448	107,464±2208	4.84±1.55	3.46 ± 0.78	2.75 ± 1.17
	>10mm		28,539 ± 4349	n.s		3.11 ± 0.45	n.s
	5 < 10mm		75,692 ± 680	48,909 ± 934		2.32 ± 0.09	3.20 ± 0.1
	5 < 2mm		47,481 ± 460	33,065 ± 603		2.23 ± 0.05	2.73 ± 0.03
	2 < 1mm		54,453 ± 2169	26,420 ± 390		2.96 ± 0.23	2.62 ± 0.01
Starch polyester blend (E3 - blue)	outside	74,969 ± 2758		59,182 ± 8	3.58 ± 0.14		3.03 ± 0.56
	>10mm		20,983 ± 240	36,339 ± 1636		2.32 ± 0.04	2.69 ± 0.19
	5 < 10mm		23,129 ± 156	n.s		2.38 ± 0.03	n.s
	5 < 2mm		n.s	n.s		n.s	n.s
	2 < 1mm		n.s	n.s		n.s	n.s
PLA (E6 - green)	outside	119,927 ± 8437	42,793 ± 3199	66,584 ± 4218	2.09 ± 0.26	1.85 ± 0.19	1.88 ± 0.18
	>10mm		n.s.	49,505 ± 393		n.s	2.07 ± 0.04
	5 < 10mm		33,167 ± 1318	n.s.		1.85 ± 0.09	n.s
	5 < 2mm		n.s.	20,620 ± 771		n.s	2.56 ± 0.13
	2 < 1mm		n.s.	n.s.		n.s	n.s

Outside: material sampled on the outside of the windrow or on the ground next to it; n.s.: quantity of recovered sample was too small to obtain a signal in SEC analysis.

The evolution of the thermal stability of the materials during the composting process was assessed by Thermo-Gravimetric Analysis (TGA) using a Mettler TGA2 apparatus (Mettler Toledo, USA). Thermal analyses were performed from 25 °C up to 800 °C at 5 °C/min under nitrogen flow (20 mL/min). An average weight of 10 mg was used each time, if possible, with regards to the available quantities, measurements were duplicated. Because this measurement was destructive but needed at least 10 mg of mass, it was carried out on mixed samples of different size classes. The data are shown in Table 12.

**Table 12:** Thermal degradation parameters of the colored materials sampled at different composting times.

Sampling time (d)		0	18	45	107	128
Starch/polyester blend (E3 – blue)	T_onset_ ( °C)	284 ± 5	250	280	265	272
	T_max_ ( °C)	392 ± 5	390	390	391	389
PHA (E9 – pink)	T_onset_ ( °C)	264 ± 5	259	253	247	255
	T_max_ ( °C)	283 ± 5	269	266	256	268
PLA (E6 – green)	T_onset_ (°C)	329 ± 5	248	229	n.r.	247
	T_max_ (°C)	352 ± 5	276	264	n.r	278

The morphological evolution of the compostable materials during the composting process was assessed by DSC (DSC1, Mettler Toledo, Switzerland) under N_2_ (flow rate 50 mL/min) using at minimum 1 mg not cleaned solid in 40 µL aluminum pans. Calibration was carried out with Indium and Zinc standards. The samples were scanned with heat-cool-heat program between – 60 and + 180 °C at 10 °C/min heating or cooling rate. Melting enthalpy (*DH_m_*) and glass transition temperature (*T_g_*) shown here were analyzed at the first heating scan. If enough material was available, measurements were done in duplicate. Because of the unknown formulation of the samples, the crystallinity degree could not be determined. The *T_g_* of the PHA samples could not be measured, because the equipment was not sensitive enough. The data are shown in Table 13.

**Table 13:** Evolution of the melting enthalpy and the glass transition temperature of the colored materials during the composting process using DSC.

Fragment size (mm)		Melting enthalpy (J/g)				Glass transition temperature (°C)			
	Time (d)	0	18	59	128	0	18	59	128
PHA (pink)	outside	82±4	80 ±1	84±1	n.s.	not measurable			
	>10 mm		90 ± 10	79 ± 17	n.s.				
	5 < 10 mm		78 ± 5	86 ± 1	60 ± 4				
	5 < 2 mm		81 ± 5	79 ± 5	75 ± 7				
	2 < 1 mm		80 ± 7	72 ± 4	54 ± 6				
Starch polyester blend (blue)	outside	64±4	62 ± 2	57 ± 8	n.s.	−33.3 ± 0.9	−33.3 ± 1.3	−32.1 ± 0.2	n.s.
	>10 mm		44 ± 12	54 ± 8	n.s.		−33.0 ± 0.8	−32.5 ± 1.1	n.s.
	5 < 10 mm		65 ± 5	56 ± 80	n.s.		−32.8 ± 1.1	−33.4 ± 0.5	n.s.
	5 < 2 mm		57 ± 15	47 ± 4	48 ± 11		−32.3 ± 0.2	−31.6 ± 0.9	−33.8 ± 0.8
	2 < 1 mm		48 ± 11	46 ± 2	65 ± 10		−33.8 ± 0.8	−32.1 ± 0.9	−32.3 ± 0.9
PLA (green)	outside	2 ± 1	30 5	n.s.	n.s.	56 ± 1	55 ± 4	50 ± 3	n.s.
	>10 mm		30	n.s.	n.s.		57	n.s.	n.s.
	5 < 10 mm		31	n.s.	n.s.		55	n.s.	n.s.
	5 < 2 mm		47	39	35		34	47	48
	2 < 1 mm		23	29	32 ± 1		20	44	49 ± 1

Outside: material sampled on the outside of the windrow or on the ground next to it; n.s.: no sample was recovered.

### 4.6 Life cycle assessment

**Fig. 2:**
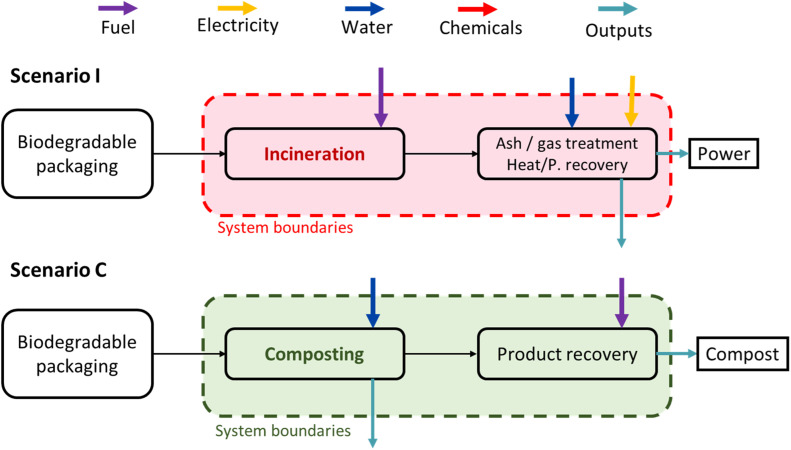
System boundaries for the environmental assessment of the incineration and composting processing of biodegradable packaging.

The life-cycle assessment (LCA) methodology was used to assess and compare the environmental performance of biodegradable packaging in two end-of-life processing treatments: industrial composting and industrial incineration. It was conducted according to the guidelines ISO 14040 and 14044 (2006) using OpenLCA v2 and the Ecoinvent® 3.0.1 database. The functional unit (FU) selected was 1 ton of packaging waste in France, composed of 70 wt% biobased material and 30 wt% fossil-based material. This composition reflects the average proportion of fossil content in the tested materials, which were not entirely biobased. The packaging waste collection was assumed to be carried out via door-to-door collection for both scenarios. Fig. 2 shows the system boundaries for each scenario.

Table 14 reports the life-cycle inventory assessment used for the environmental evaluation of biodegradable packaging under industrial composting processing. Table 15 reports the avoided impacts calculated for the replacement of inorganic fertilizer by compost and nuclear-derived electricity by incineration-derived electricity. Table 16 reports the total impact for each category, calculated by the subtraction of the avoided impacts from the generated ones.

**Table 14:** Life-cycle inventory assessment for the treatment of 1 t of biodegradable packaging by industrial composting.

Inputs for composting	Amount	Unit
Packaging waste	1	t
Diesel	7.6 × 10^–3^	kg
Electricity	47	kWh
**Outputs for composting**		
Ammonia	0.39	kg
Carbon dioxide, biogenic	278.3	kg
Carbon dioxide, fossil	78.7	kg
Carbon monoxide	0.1	kg
Compost	290	kg
Methane	3.4 × 10^–3^	kg
Nitrogen oxides	0.11	kg
VOC, volatile organic compounds	0.91	kg

**Table 15:** Avoided impacts per ton of biodegradable packaging treated by each process.

Impact category		Incineration	Composting	Unit
Abiotic depletion potential	ADP	−3.03 × 10^–5^	−5.41 × 10^–4^	kg Sb eq
Acidification potential	AP	−7.82 × 10^–2^	−4.27 × 10^–1^	kg SO_2_ eq
Eutrophication potential	EP	−3.09 × 10^–2^	−1.65 × 10^–1^	kg PO_4_^3-^ eq
Global warming (100a)	GWP	−1.61 × 10^1^	−7.25 × 10^1^	kg CO_2_ eq
Human toxicity potential	HTP	−2.07 × 10^1^	−4.08 × 10^1^	kg 1,4-DB eq
Ozone layer depletion potential	OLDP	−3.09 × 10^–5^	−1.05 × 10^–5^	kg CFC-11 eq
Photochemical oxidation potential	POP	−3.17 × 10^–3^	−1.83 × 10^–2^	kg C_2_H_4_ eq
Terrestrial ecotoxicity potential	TEP	−4.21 × 10^–2^	−4.81 × 10^–1^	kg 1,4-DB eq

**Table 16:** Total impacts (generated – avoided) per ton of biodegradable packaging treated by each process.

Impact category		Incineration	Composting	Unit
Abiotic depletion potential	ADP	−2.46 × 10^–5^	−5.37 × 10^–4^	kg Sb eq
Acidification potential	AP	−6.09 × 10^–2^	−4.16 × 10^–1^	kg SO_2_ eq
Eutrophication potential	EP	−2.49 × 10^–2^	−1.61 × 10^–1^	kg PO_4_^3-^ eq
Global warming (100a)	GWP	1.31 × 10^2^	8.46	kg CO_2_ eq
Human toxicity potential	HTP	−1.26 × 10^1^	−3.80 × 10^1^	kg 1,4-DB eq
Ozone layer depletion potential	OLDP	−2.53 × 10^–5^	−6.38 × 10^–6^	kg CFC-11 eq
Photochemical oxidation potential	POP	−2.08 × 10^–3^	−1.52 × 10^–2^	kg C_2_H_4_ eq
Terrestrial ecotoxicity potential	TEP	3.22 × 10^–1^	−4.75 × 10^–1^	kg 1,4-DB eq

## Limitations

Repeat experiments for the morphological analysis of recovered fragments at longer composting times were not always feasible due to the small quantities of remaining plastics. Destructive measurements, such as thermogravimetric analysis (TGA), could not be repeated. Additionally, size-exclusion chromatography (SEC) measurements at the end of the composting process could not be performed due to insufficient material availability.

## Ethics Statement

The authors have read and follow the ethical requirements for publication in Data in Brief and confirm that the current work does not involve human subjects, animal experiments, or any data collected from social media platforms.

## CRediT Author Statement

**Emmanuelle Gastaldi**: conceptualization, investigation, project administration, methodology, writing, reviewing and editing of draft. **Paul Greuet**: investigation, methodology, data curation. **Felipe Buendia**: conceptualization, investigation, data curation, methodology, original draft writing, reviewing and editing of draft. **Sandra Domenek**: conceptualization, investigation, project administration, data curation, original draft writing, reviewing and editing of original and final draft.

## Data Availability

Research Data GouvFull scale composting experiment of compostable packaging (Original data).

## References

[1] Gastaldi E., Buendia F., Greuet P., Benbrahim Bouchou Z., Benihya A., Cesar G. (2024). Degradation and environmental assessment of compostable packaging mixed with biowaste in full-scale industrial composting conditions. Bioresour. Technol..

[2] Kale G., Auras R., Singh S.P. (2006). Degradation of commercial biodegradable packages under real composting and ambient exposure conditions. J. Polym. Environ..

[3] Musiol M., Sikorska W., Adamus G., Janeczek H., Kowalczuk M., Rydz J. (2016). (Bio)degradable polymers as a potential material for food packaging: studies on the (bio)degradation process of PLA//(R,S)-PHB rigid foils under industrial composting conditions. Eur. Food Res. Technol..

[4] Musiol M., Sikorska W., Adamus G., Janeczek H., Richert J., Malinowski R. (2016). Forensic engineering of advanced polymeric materials. Part III - Biodegradation of thermoformed rigid PLA packaging under industrial composting conditions. Waste Manag..

[5] Zhang H., McGill E., Gomez C.O., Carson S., Neufeld K., Hawthorne I. (2017). Disintegration of compostable foodware and packaging and its effect on microbial activity and community composition in municipal composting. Int. Biodeterior. Biodegrad..

[6] Klauss M., Bidlingmaier W. (2004). Pilot scale field test for compostable packaging materials in the City of Kassel, Germany. Waste Manag..

[7] Greene J. (2007). Biodegradation of compostable plastics in green yard-waste compost environment. J. Polym. Environ..

[8] Leppanen I., Vikman M., Harlin A., Orelma H (2020). Enzymatic degradation and pilot-scale composting of cellulose-based films with different chemical structures. J. Polym. Environ..

[9] Kawashima N., Yagi T., Kojima K. (2021). Pilot-scale composting test of polylactic acid for social implementation. Sustainability.

[10] Salomez M., George M., Fabre P., Touchaleaume F., Cesar G., Lajarrige A. (2019). A comparative study of degradation mechanisms of PHBV and PBSA under laboratory-scale composting conditions. Polym. Degrad. Stab..

[11] Domenek S., Feuilloley P., Gratraud J., Morel M.H., Guilbert S. (2004). Biodegradability of wheat gluten based bioplastics. Chemosphere.

